# An extension of Phase Linearity Measurement for revealing cross frequency coupling among brain areas

**DOI:** 10.1186/s12984-019-0615-8

**Published:** 2019-11-07

**Authors:** Pierpaolo Sorrentino, Michele Ambrosanio, Rosaria Rucco, Fabio Baselice

**Affiliations:** 1 Department of Engineering, University of Naples Parthenope, Naples, Italy; 2 Department of Science and Technology, University of Naples Parthenope, Naples, Italy

**Keywords:** Brain connectivity, Phase coupling, Cross frequency synchronization, Connectivity metrics

## Abstract

**Background:**

Brain areas need to coordinate their activity in order to enable complex behavioral responses. Synchronization is one of the mechanisms neural ensembles use to communicate. While synchronization between signals operating at similar frequencies is fairly straightforward, the estimation of synchronization occurring between different frequencies of oscillations has proven harder to capture. One specifically hard challenge is to estimate cross-frequency synchronization between broadband signals when no a priori hypothesis is available about the frequencies involved in the synchronization.

**Methods:**

In the present manuscript, we expand upon the phase linearity measurement, an iso-frequency synchronization metrics previously developed by our group, in order to provide a conceptually similar approach able to detect the presence of cross-frequency synchronization between any components of the analyzed broadband signals.

**Results:**

The methodology has been tested on both synthetic and real data. We first exploited Gaussian process realizations in order to explore the properties of our new metrics in a synthetic case study. Subsequently, we analyze real source-reconstructed data acquired by a magnetoencephalographic system from healthy controls in a clinical setting to study the performance of our metrics in a realistic environment.

**Conclusions:**

In the present paper we provide an evolution of the PLM methodology able to reveal the presence of cross-frequency synchronization between broadband data.

## Introduction

In order to perform complex behavior, brain areas must coordinate to process information coherently [1]. To do so, brain regions tend to entrain each others activity [2]. Hence, the framework of synchronization has been exploited to successfully capture such a phenomenon. Most of the work that has been done so far has focused on the case of two signals oscillating at the same frequency. However, communication also occurs between brain areas operating at different frequencies [3, 4]. So far, this cross-frequency synchronization occurs in the brain through two distinct mechanisms. In one case, the amplitude of a signal modulates the phase of a second signal. This mechanism is normally referred to as phase-amplitude coupling [5]. The second mechanism occurs when *n* oscillations of the first signal are synchronized with *m* oscillations of the second signal [6, 7]. This mechanism is normally referred to as “n:m synchronization”, and plays a critical role in the brain, since it is the only known way by which two brain areas can communicate at the time scale of the faster area [8–10].

Given that fast communication in the brain is crucial to obtain efficient behavioural responses, a number of metrics have been designed in order to quantify the occurrence of this phenomenon, such as the bispectrum and the bicoherence [11]. In fact, if one has an a priori hypothesis on the frequencies between which the synchronization might be occurring (or, somewhat equivalently, if the signals are narrowband), estimating n:m synchronization is fairly straightforward. However, when dealing with electroencephalography (EEG) or magnetoencephalography (MEG), one deals with broadband signals and, if no hypothesis is available about the frequency of the components that might be synchronous, it is not possible to use the available metrics (unless one tries the brute-force approach, i.e. all the possible combinations of frequencies [12]).

For example, when one deals with resting-state data, it is not known if n:m cross-frequency synchronization is occurring and, if it is, where and between which frequencies. In the current manuscript, we modify an iso-frequency metric designed by our group, the phase linearity measurement (PLM) [13], applying the same principles in order to determine, from broadband data, if any component from the signals is synchronized with others and, if so, the frequencies involved in the coupling. The underlying idea, as explained in the methods, lies in the study of the shape of the interferometric spectrum computed comparing the two signals. After introducing the metric, we first evaluate its properties on a model based on Gaussian processes. Furthermore, we test the performance of the metric on real data, as a proof-of-concept of the applicability of our approach to real MEG data.

## Methods

The phase linearity measurement (PLM) is able to effectively measure the iso-frequency functional connectivity between brain areas by analyzing the signals synchronization [13]. Given the time series related to two brain regions acquired by an EEG or a MEG system, the PLM algorithm requires as first step the computation of their analytic signals, followed by the extraction of the phase difference. Subsequently, a frequency analysis is carried out by means of the fast Fourier transform (FFT). More in details, the power spectrum of the phase difference is computed, and its integral in a narrow bandwidth [-B, B] is measured. The assumption is that if the two brain regions are exchanging information, a certain level of phase synchronization will occur between their signals. Such a synchronization will produce a power spectrum concentrated around the zero frequency. On the contrary, in case of no synchronization, the power spectrum will spread over a wide range of frequencies. Therefore, the computation of the amount of power gathered at the very low frequencies (i.e. the [-B, B] range) is an effective measurement of the synchronization between sources.

From a mathematical point of view, the PLM value is computed according to:

1 $$ PLM=\frac{\int_{-B}^{B} S_{Z}(f) \mathrm{d}f}{\int_{-\infty}^{\infty} S_{Z}(f) \mathrm{d}f}\,.   $$

In the above equation, *B* is the unilateral integration bandwidth and *S*_*Z*_(*f*) is the power spectrum of the phase difference, i.e.:

2 $$ S_{Z}(f)=\left| \int_{0}^{T} e^{i (\sphericalangle x(t))} e^{-i (\sphericalangle y(t))} e^{-i2\pi f t} \mathrm{d}t \right|^{2}\,,   $$

where *x*(*t*) and *y*(*t*) are two acquired signals, the functional $\sphericalangle $ extract the phase term and *T* is the observation period.

**Fig. 1:**
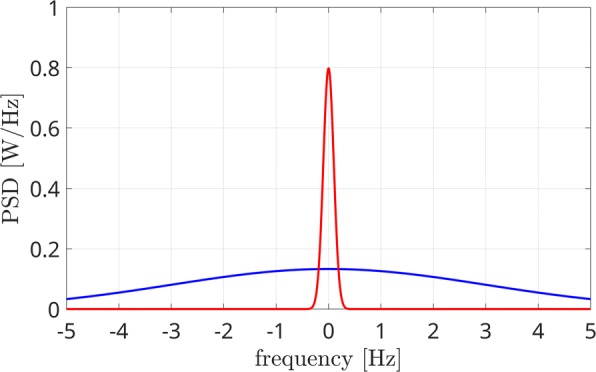
Power spectrum of the phase difference in case of two coupled (red line) and uncoupled (blue line) sources

In Fig. 1, two power spectra are reported in case of synchronized (red line) and unsynchronized (blue line) sources. The first one is characterized by a high percentage of power gathered around *f*=0, while the latter not. By measuring how the area of the two curves is concentrated close to zero, we can measure the synchronization and thus the phase connectivity between brain sources.

The PLM measurement, as presented in [13], is effective in measuring brain connectivity in the iso-frequency case, i.e. when the two signals are within the same frequency bandwidth. In case two brain regions are exchanging information but they are working at different frequencies, Eq. (1) is no more effective in measuring connectivity. This happens because the PLM assumption that the power spectrum *S*_*Z*_(*f*) is concentrated around zero in case of coupling is no more valid. More in detail, it will still be characterized by a narrow peak, but it will be centered at the frequency *Δf*=*f*_*x*_−*f*_*y*_, i.e. the difference between the oscillation frequencies of the two brain sources. For example, if two coupled sources are considered, with the first one producing a signal in the alpha band (*f*_*x*_=10 Hz) and the second one in the beta band (*f*_*y*_=19 Hz), the peak of power spectrum will be located at *Δf*=−9 Hz. In other words, the red line of Fig. 1 will be horizontally shifted of a quantity that depends on the difference between the signals central frequencies, which is 9 Hz in the considered example.

In order to correctly measure cross-frequency connectivity (CFC), a proper strategy has to be defined for handling such a situation. Our solution consists in identifying the position of the global maximum of the power spectrum function *S*_*Z*_(*f*), namely *f*_*M*_, and in integrating over an interval centered in that position, i.e. in the [ *f*_*M*_−*B,f*_*M*_+*B*] range. Therefore, the PLM expression of Eq. (1) is updated to:

3 $$ PLM_{CFC}=\frac{\int_{f_{M}-B}^{f_{M}+B} S_{Z}(f) \mathrm{d}f}{\int_{-\infty}^{\infty} S_{Z}(f) \mathrm{d}f}\,.   $$

**Fig. 2:**
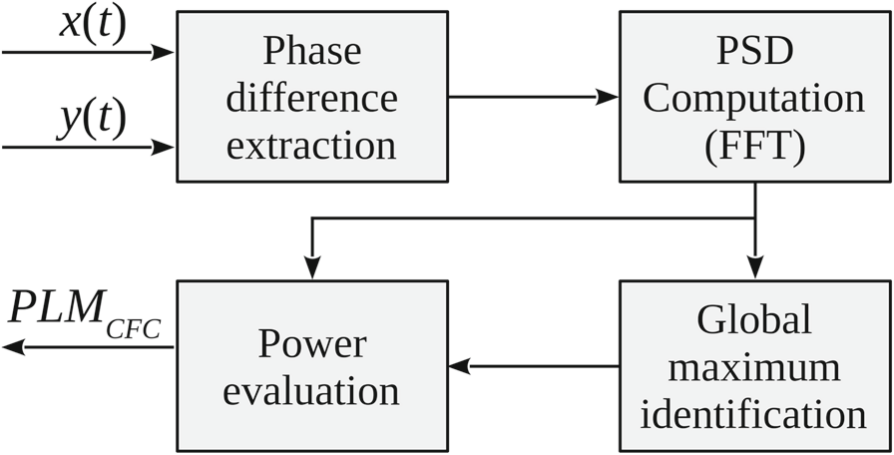
The processing chain of the proposed *PLM*_*CFC*_ algorithm

For instance, assuming that the maximum is located at -9 Hz and a unilateral integration bandwidth *B*=1 Hz is employed, the integral at the numerator will be between -10 and -8 Hz. The processing steps required by the proposed *PLM*_*CFC*_ algorithm are reported in Fig. 2.

We have to underline that the algorithm requires the identification of the peak position of the function *S*_*Z*_(*f*), i.e. *f*_*M*_. Such estimation can be easily carried out from the measured signals, nevertheless the knowledge of *f*_*M*_ gives us only information about the difference between the frequencies of the two signals involved in the coupling, but the two frequencies are not identified. In other words, the *PLM*_*CFC*_ algorithm is able to measure the amount of information exchanged (the connectivity), also in case of cross-frequency coupling, but it is not able to identify the frequency bandwidths involved in such a communication.

## Experimental results

Two analysis have been conducted for evaluating the performance of the proposed method in measuring cross-frequency connectivity. More in detail, a simulated scenario based on realizations of coupled Gaussian random processes has been set up for simulating cross-frequency connectivity. Moreover, real data have been exploited for the validation of the approach.

### Simulated data

**Fig. 3:**
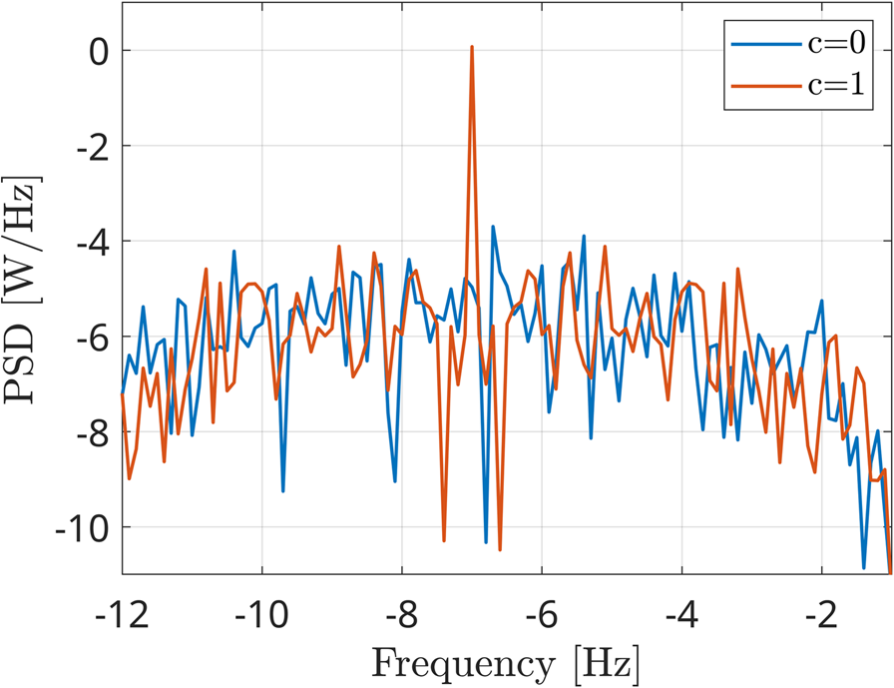
Two interferometric spectra (PSD) in case of a coupled pair of sources at different frequencies (red line) and in case of uncoupled signals (blue line)

Several realizations of one couple of white Gaussian random processes have been generated in a Monte Carlo simulation. The frequency range has been set equal to [0.5, 50] Hz. Subsequently, a correlation varying within the [0, 1] range has been applied. In order to simulate cross-frequency correlation, the second signal has been frequency-shifted of 7 Hz by multiplying it for a complex phasor. As example, two interferometric spectra are reported in Fig. 3, related to the independent (blue line) and correlated (red line) signals. Note that the red line shows a peak centered at -7 Hz, as this frequency shift has been considered. As expected, this power peak appears only in case of non-zero correlation, being an indicator of the cross-frequency coupling between sources.

**Fig. 4:**
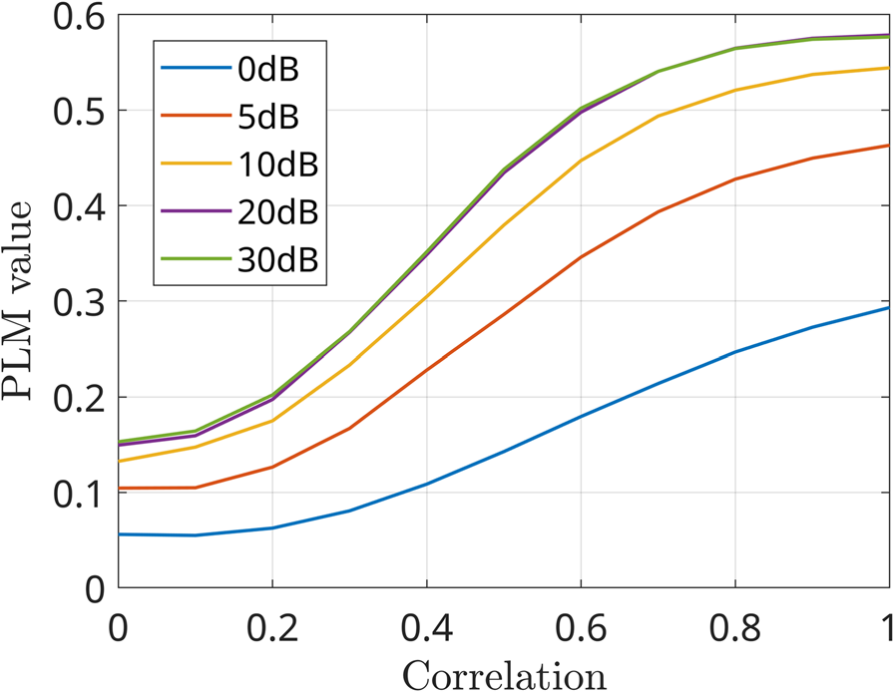
Measured connectivity as a function of correlation of the Gaussian processes in case of different SNR values

**Fig. 5:**
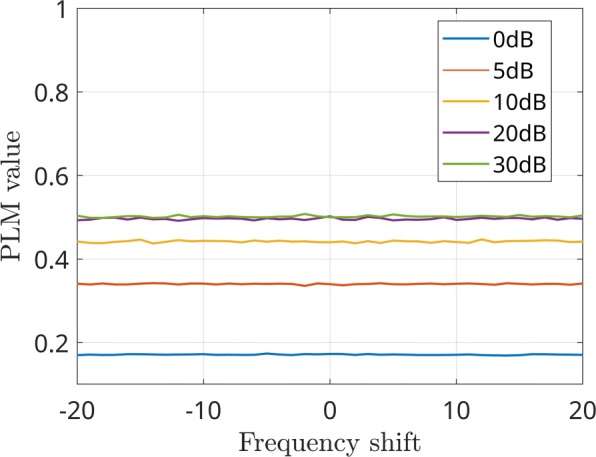
Measured connectivity as a function of frequency shift in case of different SNR values

The first analysis aims at evaluating the effectiveness of the *PLM*_*CFC*_ algorithm in measuring the connectivity in the cross-frequency case. The curves reported in Fig. 4 show that the *PLM*_*CFC*_ value increases with the correlation between Gaussian processes, as expected. Moreover, although the maximum values are related to the noise level, the curves in case of different SNR values have a very similar behavior, showing that the sensitivity of the measurement is not affected. A second analysis aims at evaluating the performance in case of different values of a frequency-shift between sources. Some results are reported in Fig. 5 for SNR values between 0dB and 30dB. Globally, it can be stated that the *PLM*_*CFC*_ values are minimally affected by the difference between the two involved frequencies.

### Real data

The Magnetoencephalographic data of a healthy subject acquired by the system built by the Italian national research council (CNR) and located in Naples, Italy, has been adopted for testing the proposed methodology. The acquisition was performed in resting state condition for a period of 150 seconds. The data has been sampled at 1024 Hz and cleaned from the artifacts by visual inspection of trained experts. The linearly constrained minimum variance (LCMV) beamformer has been implemented for reconstructing the signals in the source domain. For this step, data has been re-sampled at 512 Hz and the 116 region AAL atlas has been considered. The processing has been done in a Matlab environment exploiting the Fieldtrip toolbox. More details about the acquisition pipeline can be found in [14].

We focused our analysis on the following four source pairs, as they were found illustrative of different types of connectivity:

- left precentral gyrus (n. 14 of the AAL) and the left middle frontal gyrus (n. 8 of the AAL);
- right inferior parietal lobe (n. 57 of the AAL) and the right gyrus rectus (n. 40 of the AAL);
- left superior occipital gyrus (n. 22 of the AAL) and the left superior frontal gyrus (n. 7 of the AAL);
- right middle occipital gyrus (n. 23 of the AAL) and the paracentral lobule (n. 13 of the AAL).

**Fig. 6:**
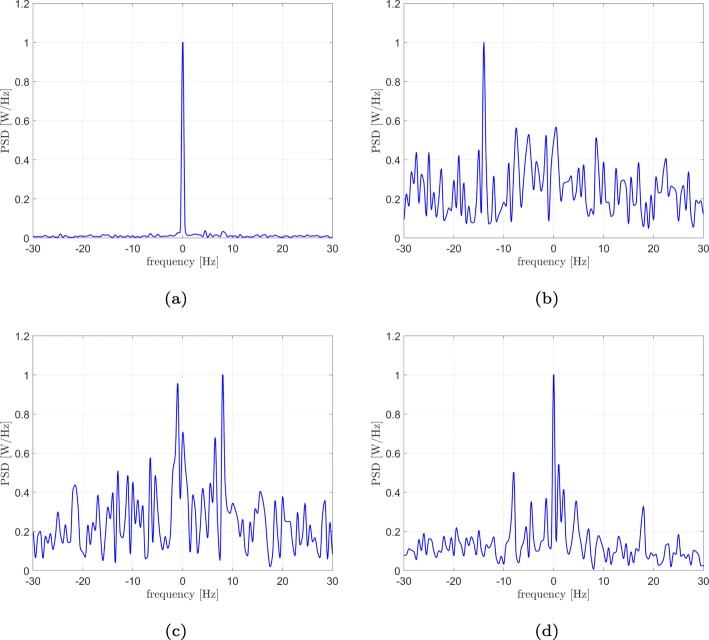
Real data results: power spectra of the interferometric signals of different pairs. Between sources 14 and 8 of the AAL, only an iso-frequency coupling (peak at 0 Hz) is present (**a**), sources 57 and 40, only a cross-frequency coupling (peak at -14 Hz) is present (**b**), sources 22 and 7, both iso and cross-frequency coupling (peaks at 0 and 8 Hz) are present (**c**), sources 23 and 13, in addition to the iso-frequency peak, several cross-frequency couplings (peaks at -8, 0 and 18 Hz) are present (**d**)

For each of them, the interferometric signal and its PSD have been computed. In particular, the power spectra are reported in Fig. 6 in a linear scale. We chose such sources in order to show different types of communications between brain areas. More in detail, the analysis of sources 14 and 8 (Fig. 6a) found that only the iso-frequency coupling is responsible for their mutual communications with a power percentage of 41% of the iso-frequency peak with respect to the total power (the fraction of power was evaluated by employing a 1-Hz unilateral bandwidth centred in the peak of interest). Figure 6b shows that the cross-frequency coupling is preeminent in case of regions 57 and 40 with a power percentage equal to 4.34%. Regions 22 and 7 (Fig. 6c) make use of both iso and cross-frequency coupling, with power percentages of 6.5% and 4.5%, respectively, while sources 23 and 13 (Fig. 6d) show three communication channels (peaks at -8, 0 and 18 Hz with power percentages equal to 4.4%, 7.6% and 3.34%, respectively).

## Discussion

In this manuscript, we propose a novel procedure to reveal the presence of n:m synchronization between brain areas, starting from broadband signals such as those derived from electroencephalography or magnetoencephalography.

The proposed metric is based on the analysis of the shape of the interferometric spectrum of the two signals. This approach is an evolution of a previously published metrics, the phase linearity measurement (PLM), that uses the interferometric spectrum to detect the presence of iso-frequency synchronization [13]. In brief, the proposed approach explores the interferometric spectrum, looking for the presence of power peaks at frequencies different from 0 Hz (related to the iso-frequency coupling). If more peaks are present, this implies that synchronization between signal components at different frequencies must be occurring. In the case of Gaussian random processes, signals with a realistic, broad frequency range were used for testing the approach.

Firstly, we show that, when introducing a correlation, this is correctly detected in the interferometric spectrum by the appearance of a peak, located at a frequency value equal to the difference between the frequencies of the synchronized components. Importantly, we show that the intensity of the peak grows monotonically with the strength of the correlation between the two signals. Furthermore, we show that our procedure is robust to realistic levels of noise. This is important for the possibility of applying this kind of estimate to EEG and MEG data, that are normally located in very noisy environments, such as hospitals and clinics.

With regard to the analysis of real data, we used source-reconstructed MEG signals that have been recorded from healthy subjects from the MEG facility in Naples, Italy. As expected from our previous work, nearly in all cases the most prominent peak in the interferometric spectrum was the one occurring at 0 Hz [13]. This is not surprising, taking into account the importance of iso-frequency synchronization in the human brain [2]. However, it is important to notice that some regions showed a different pattern in the synchronization. In fact, a subset of regions showed a second peak, either alone, or co-occurring with the peak centered in 0. This finding points towards the idea that, even in resting-state, cross-frequency synchronization is taking place and is contributing to the coherent unfolding of brain activity [3]. Furthermore, this data also shows that cross-frequency coupling is not an ubiquitous phenomenon but it occurs in a spatially constrained fashion (i.e. it preferentially happens in specific areas).

Finally, our findings show that, at least in some cases, the multiple components contributing to the reconstructed signal from an area can be independent from each other and, possibly, have a different biological meaning and interpretation.

## Conclusions

The present work introduces a robust methodology for the estimation of phase-to-phase, cross-frequency synchronization from broadband data, without any a priori hypothesis about the frequency of the synchronized components. Our methodology provides an important tool to understand how the activities in different frequency bandwidths in the resting state brain obtain coherent, synchronized activity. Furthermore, optimal resilience against noise will allow the use of this procedure also in patients, that typically display noisy acquisitions, helping elucidating disease mechanisms from a whole-brain perspective.

## Data Availability

The dataset analysed during the current study is available from the corresponding author on reasonable request.

## Abbreviations

AAL: Automated anatomical labeling
CFC: Cross frequency coupling
CNR: Italian national research council
EEG: Electroencephalography
FFT: Fast fourier transform
LCMV: Linearly constrained minimum variance
MEG: Magnetoencephalography
PLM: Phase linearity measurement
PSD: Power spectral density
SNR: Signal to noise ratio
